# Causal relationship between type 1 diabetes mellitus and mycoses: a Mendelian randomization study

**DOI:** 10.3389/fmed.2024.1408297

**Published:** 2024-06-14

**Authors:** Xiaolan Chen, Chen Chen, Mingyan Wu, Shanmei Wang, Hongbin Jiang, Zhe Li, Yuetian Yu, Bing Li

**Affiliations:** ^1^Department of Emergency, Shanghai Pulmonary Hospital, School of Medicine, Tongji University, Shanghai, China; ^2^Department of Critical Care Medicine, Renji Hospital, School of Medicine, Shanghai Jiao Tong University, Shanghai, China; ^3^Department of Respiratory and Critical Care Medicine, Shanghai Pulmonary Hospital, School of Medicine, Tongji University, Shanghai, China

**Keywords:** diabetes mellitus type 1, mycoses, candidiasis, Mendelian randomization analysis, infections

## Abstract

**Background:**

Type 1 diabetes mellitus (T1DM) is frequently associated with various infections, including mycoses; however, the direct link between T1DM and fungal infections remains under-researched. This study utilizes a Mendelian randomization (MR) approach to investigate the potential causal relationship between T1DM and mycoses.

**Methods:**

Genetic variants associated with T1DM were sourced from the European Bioinformatics Institute database, while those related to fungal infections such as candidiasis, pneumocystosis, and aspergillosis were obtained from the Finngen database, focusing on European populations. The primary analysis was conducted using the inverse variance weighted (IVW) method, with additional insight from Mendelian randomization Egger regression (MR-Egger). Extensive sensitivity analyses assessed the robustness, diversity, and potential horizontal pleiotropy of our findings. Multivariable Mendelian randomization (MVMR) was employed to adjust for confounders, using both MVMR-IVW and MVMR-Egger to evaluate heterogeneity and pleiotropy.

**Results:**

Genetically, the odds of developing candidiasis increased by 5% in individuals with T1DM, as determined by the IVW method (OR = 1.05; 95% CI 1.02–1.07, *p* = 0.0001), with a Bonferroni-adjusted *p*-value of 0.008. Sensitivity analyses indicated no significant issues with heterogeneity or pleiotropy. Adjustments for confounders such as body mass index, glycated hemoglobin levels, and white blood cell counts further supported these findings (OR = 1.08; 95% CI:1.03–1.13, *p* = 0.0006). Additional adjustments for immune cell counts, including CD4 and CD8 T cells and natural killer cells, also demonstrated significant results (OR = 1.04; 95% CI: 1.02–1.06, *p* = 0.0002). No causal associations were found between T1DM and other fungal infections like aspergillosis or pneumocystosis.

**Conclusion:**

This MR study suggests a genetic predisposition for increased susceptibility to candidiasis in individuals with T1DM. However, no causal links were established between T1DM and other mycoses, including aspergillosis and pneumocystosis.

## 1 Introduction

Mycoses play a critical role in escalating morbidity and worsening outcomes for certain vulnerable populations, including patients with hematologic and solid organ malignancies, as well as those in critical care settings. The 2008 guidelines clarify the host factors linked with invasive pulmonary mycoses, highlighting conditions that severely weaken the immune system. These include recent occurrences of neutropenia, hematopoietic stem cell transplants, and Acquired Immune Deficiency Syndrome (AIDS) (1). The 2019 update to these guidelines incorporated additional host factors such as solid organ transplantation and the use of B-cell immunosuppressants (2). Despite these detailed guidelines, there are notable limitations in their scope concerning host factors. Approximately 30 to 70% of patients with invasive pulmonary mycoses do not fit the classical host factor criteria specified in the guidelines. In individuals with Type 1 diabetes mellitus (T1DM), compromised immune function related to abnormalities in purine metabolism, chemotactic inflammation, and macrophage activity may increase their vulnerability to fungal infections. For instance, a study involving 1,192 patients newly diagnosed with acute myeloid leukemia identified diabetes mellitus as a risk factor for developing invasive aspergillosis, although it did not show a similar risk for invasive candidiasis (3). Similarly, a retrospective study of deep fungal infections in elderly individuals found that diabetic patients were prone to developing invasive candidiasis, while invasive aspergillosis was less prevalent (4). Despite the established connection between diabetes and bacterial infections, the association between T1DM and mycoses remains a less explored area, presenting an intriguing path for future study (5).

Type 1 diabetes mellitus (T1DM) is a chronic autoimmune disorder characterized by the destruction of pancreatic islet beta cells. This leads to insulin deficiency and elevated blood glucose levels (6). While extensive scientific studies have elucidated the metabolic challenges associated with T1DM, recent inquiries have shed light on a growing concern: the heightened susceptibility of T1DM individuals to mycoses (7). Although diabetes is known to exacerbate fungal infections and lead to poor prognosis, there is limited evidence based on evidence-based medicine. Much of the literature is currently focused on the relationship between type 2 DM and fungal infections (8). As there are fewer retrospective and prospective clinical studies to demonstrate the relationship between type 1 diabetes and fungal infections, we used a Mendelian randomization study to fill an important knowledge gap and possibly the casual relationship between mycoses and T1DM.

Mendelian Randomization (MR) stands as a valuable analytical tool frequently employed in epidemiology to discern causality, leveraging the principles of Mendelian independent assortment. Establishing a plausible causal pathway for MR is paramount. Previous observational research has highlighted numerous associations between T1DM and mycoses, suggesting a potential correlation between these conditions. This study employs a comprehensive MR analysis to uncover the potential causal link between T1DM and mycoses. Furthermore, it utilizes the multivariable MR (MVMR) method to explore confounding factors such as glycated hemoglobin and blood immune cells in the interplay between T1DM and mycoses.

## 2 Methods

**Figure 1:**
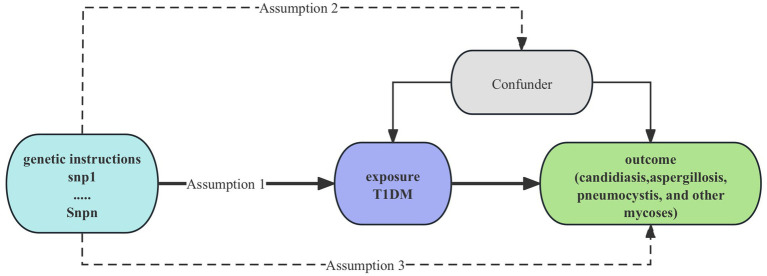
Study design of causality Between T1DM and mycoses. T1DM, type 1 diabetes mellitus; snp, single-nucleotide polymorphisms.

Univariable MR analysis was performed to detect the causal link between T1DM and mycoses in our study (9). MR utilizes genetic variation as a proxy for risk factors; therefore, the instrumental variables (IVs) used in causal inference must adhere to three critical assumptions: 1. Hypothesis of association: a significant correlation exists between single-nucleotide polymorphisms (SNPs) and exposure factors. 2. Hypothesis of independence: SNPs are not associated with confounding factors. 3. Assumption of exclusivity: The impact of SNPs on outcomes is mediated exclusively through exposure factors. The study design is shown on Figure 1. To investigate the direct influence of T1DM on mycoses, we conducted a multivariable Mendelian randomization (MR) analysis, which extends beyond univariable MR by enabling the identification of causal effects involving multiple risk factors concurrently. This paper adheres to the STROBE-MR principle (10).

### 2.1 Data source

Genetic variations linked to T1DM were sourced from the European Bioinformatics Institute (EBI) database (11). Data on candidiasis, aspergillosis, pneumocystis, and other mycoses were acquired from the FinnGen Consortium (12), involving participants. Adjustment variables such as BMI, HbA1c, lymphocyte count, monocyte count, neutrophil count, CD4 regulatory T cell, CD4+ T cell, CD8+ T cell, Nature Kill (NK) T cell count, and B cell absolute count were also sourced from the EBI database.

### 2.2 IVs selection

The threshold of instrumental variables (IVs) was established at 5 × 10^^−6^ (13, 14). To filter out SNPs with a linkage disequilibrium (LD) threshold of r^^2^ < 0.001 within a 10,000 kb range, the clumping procedure was implemented using R software version 4.2.0. The F statistic was utilized as a key indicator of the statistical robustness of the IVs. Calculation of the F statistic was based on the formula F = R^2(N−K−1)/K (1−R^2) (15).

### 2.3 Statistical analysis

The inverse variance weighted (IVW) method was used as the main approach, complemented by additional methods such as MR-Egger, weighted median, simple mode, and weighted mode (16). We calculated odds ratios (OR) along with their 95% confidence intervals (CI), considering statistical significance to be present at *p* < 0.05. We use the Bonferroni method to correct the *p*-value. SNP heterogeneity was assessed using Cochran’s Q statistic and corresponding *p*-values to test for horizontal pleiotropy among the selected IVs. To assess heterogeneity, we employed both the Mendelian randomization pleiotropy residual sum and outlier (MR-PRESSO) method and the MR-Egger approach (9, 17). Funnel plots showed the robustness of the correlation and absence of heterogeneity. MVMR analyses were conducted to adjust for confounding factors (17) including BMI (18), HbA1c, neutrophil count, lymphocyte count, monocyte count, and lymphocyte classification. MVMR-IVW and MVMR-Egger were performed to detect heterogeneity and potential pleiotropy (19). HbA1c was selected as a confounding variable based on research suggesting that elevated blood sugar levels, rather than diabetes alone, significantly increase morbidity and mortality from infectious diseases. Patients with diabetes mellitus exhibit reduced neutrophil chemotaxis, phagocytosis, intracellular bactericidal activity, and limited lymphocyte activation, potentially increasing susceptibility to fungal infections (20). Therefore, we corrected for confounding factors such as neutrophil count, lymphocyte count, monocyte count, and lymphocyte classification (including CD4 regulatory T cell, CD8+ T cell, CD4+ CD8dim T cell, Natural Killer T cell, and B cell Absolute Count). All analyses were conducted as two-sided tests using the Two Sample MR package (version 0.5.7) within R software (version 4.2.0).

## 3 Results

### 3.1 Exploration of the causal effect of T1DM onset on candidiasis

**Figure 2:**
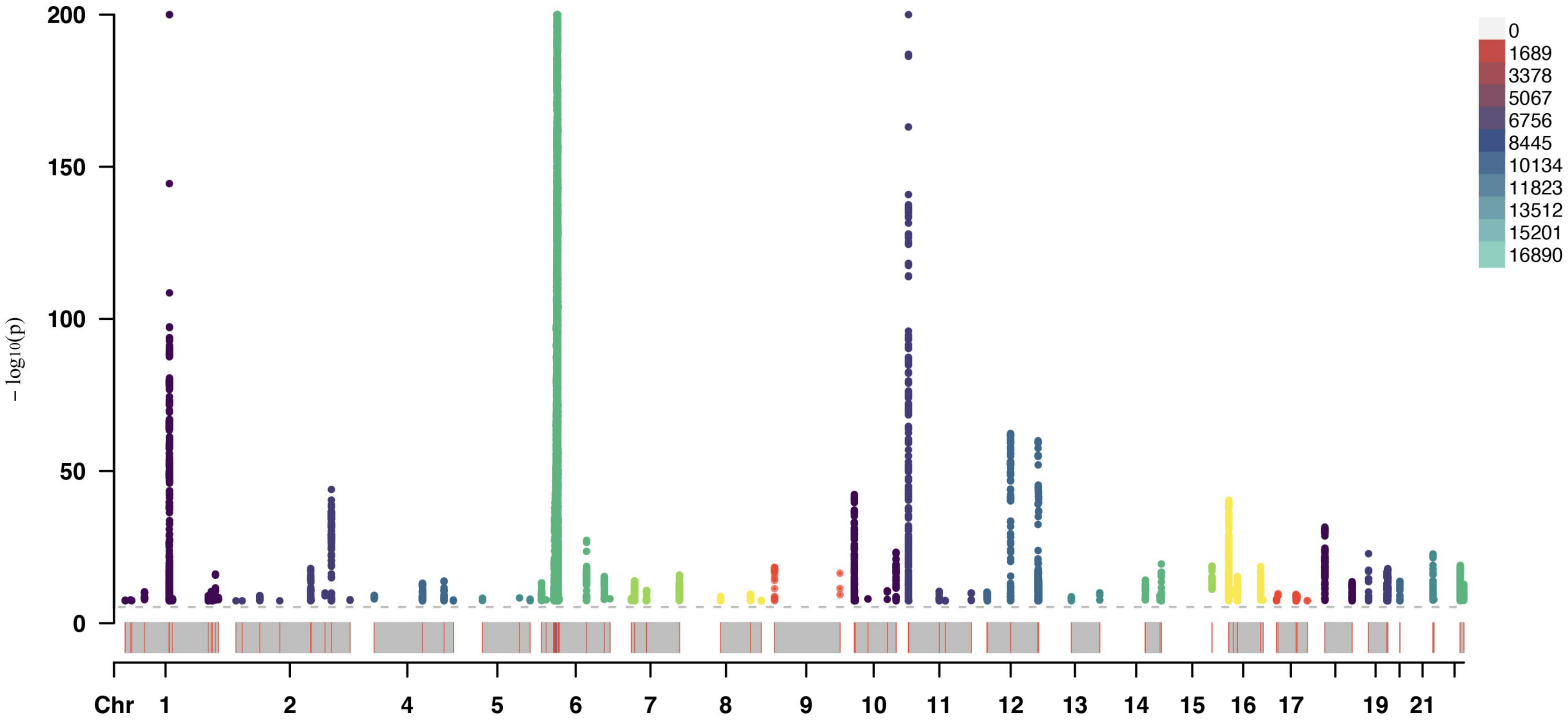
GWAS information of T1DM. GWAS, genome-wide association study.

**Figure 3:**
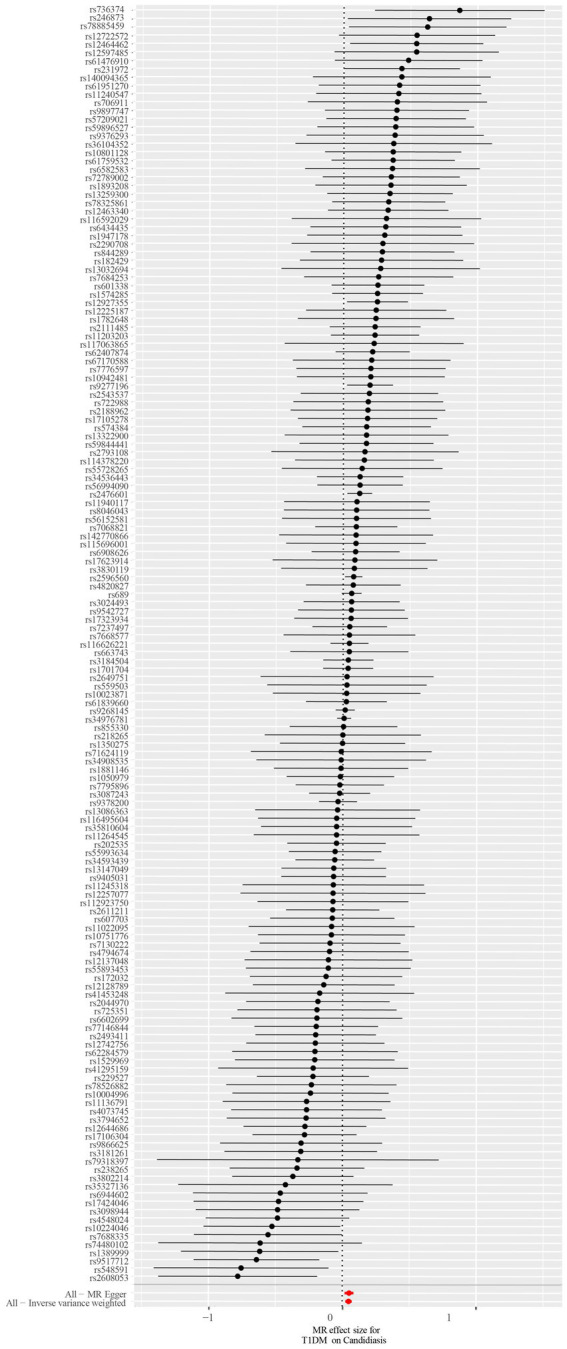
The forest plot depicts the impact of each SNP on candidiasis. Black lines represent the effect of an individual SNP, while a red line signifies the causal estimation using all ivs. If the solid line is positioned entirely to the left of 0, it suggests that T1DM may reduce the likelihood of candidiasis based on this SNP. Conversely, it implies that T1DM might potentially increase the risk of candidiasis. An intersection of the solid line with 0 indicates an insignificant result. SNP, single-nucleotide polymorphisms; ivs, instrumental variables; T1DM, type 1 diabetes mellitus.

**Figure 4:**
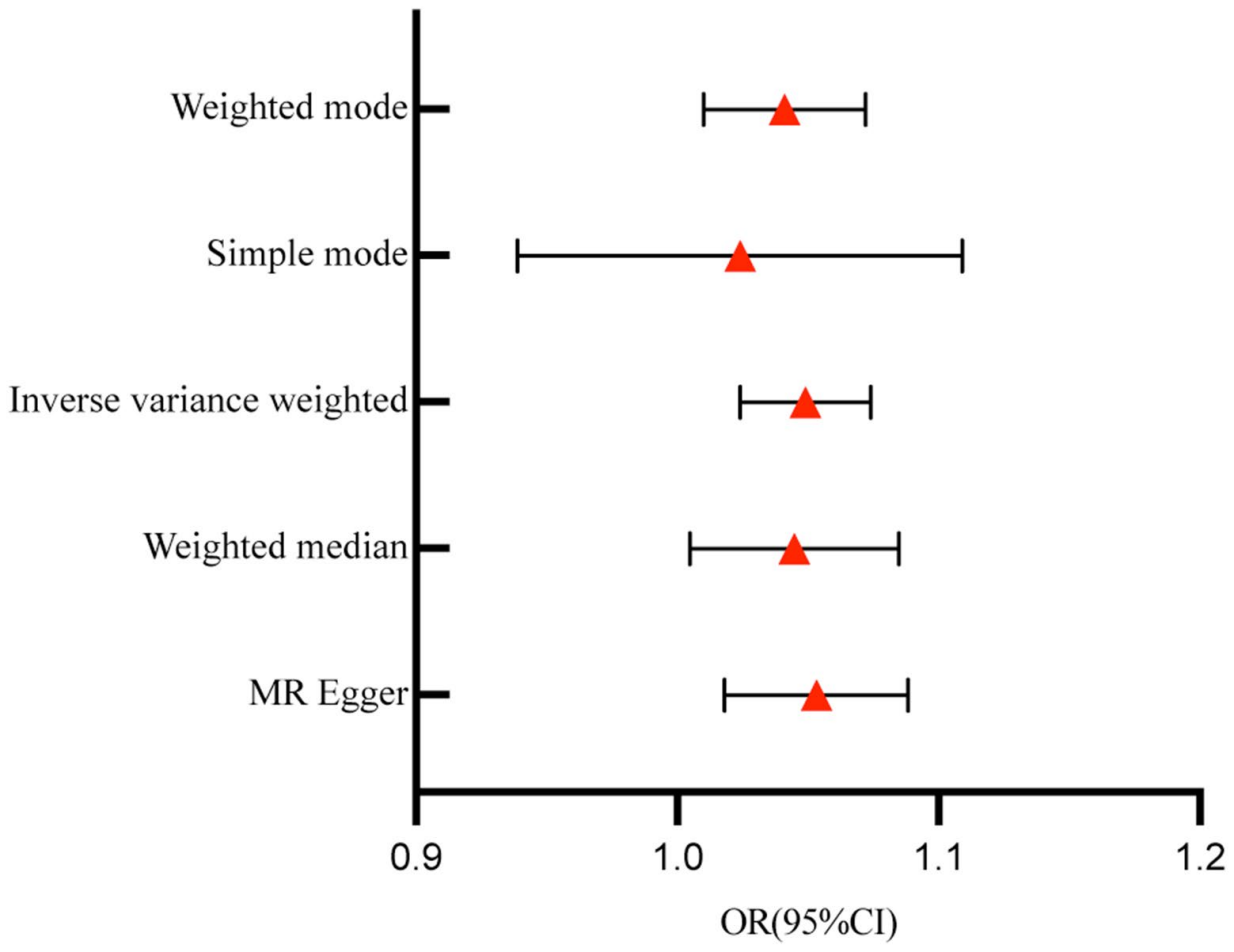
Forest plot for the effect of T1DM on candidiasis. T1DM, type 1 diabetes mellitus; OR, odds ratios; CI, confidence intervals.

**Figure 5:**
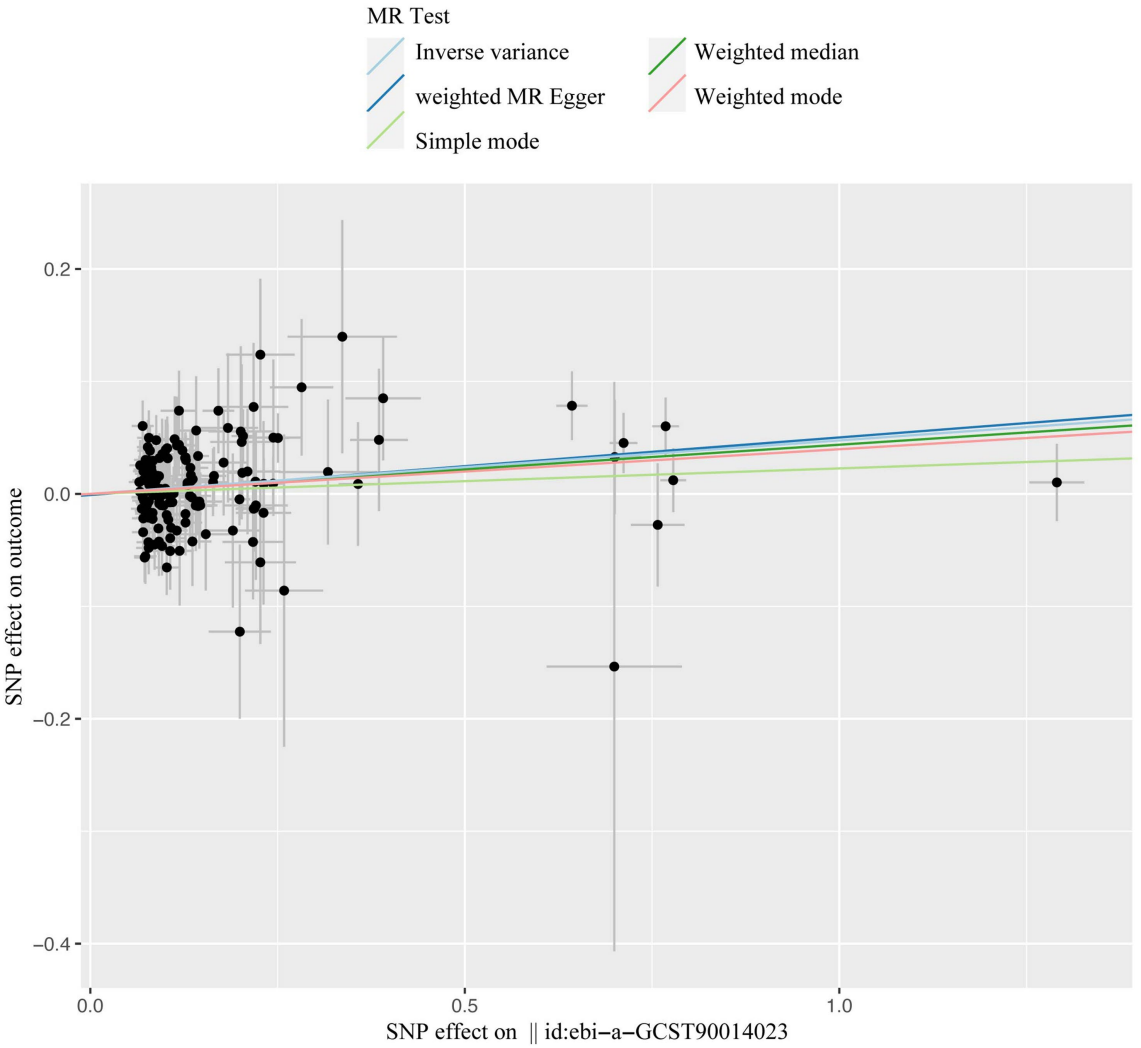
The scatter plot of casual effect of T1DM on candidiasis. Black data points represent ivs, with the horizontal axis indicating the influence of SNPs on T1DM, and the vertical axis indicating the influence of SNPs on candidiasis occurrence. Colored lines depict the results of MR analysis utilizing five different methods. T1DM, type1 diabetes mellitus; ivs, instrumental variables; SNP, single-nucleotide polymorphism; MR, Mendelian randomization.

**Figure 6:**
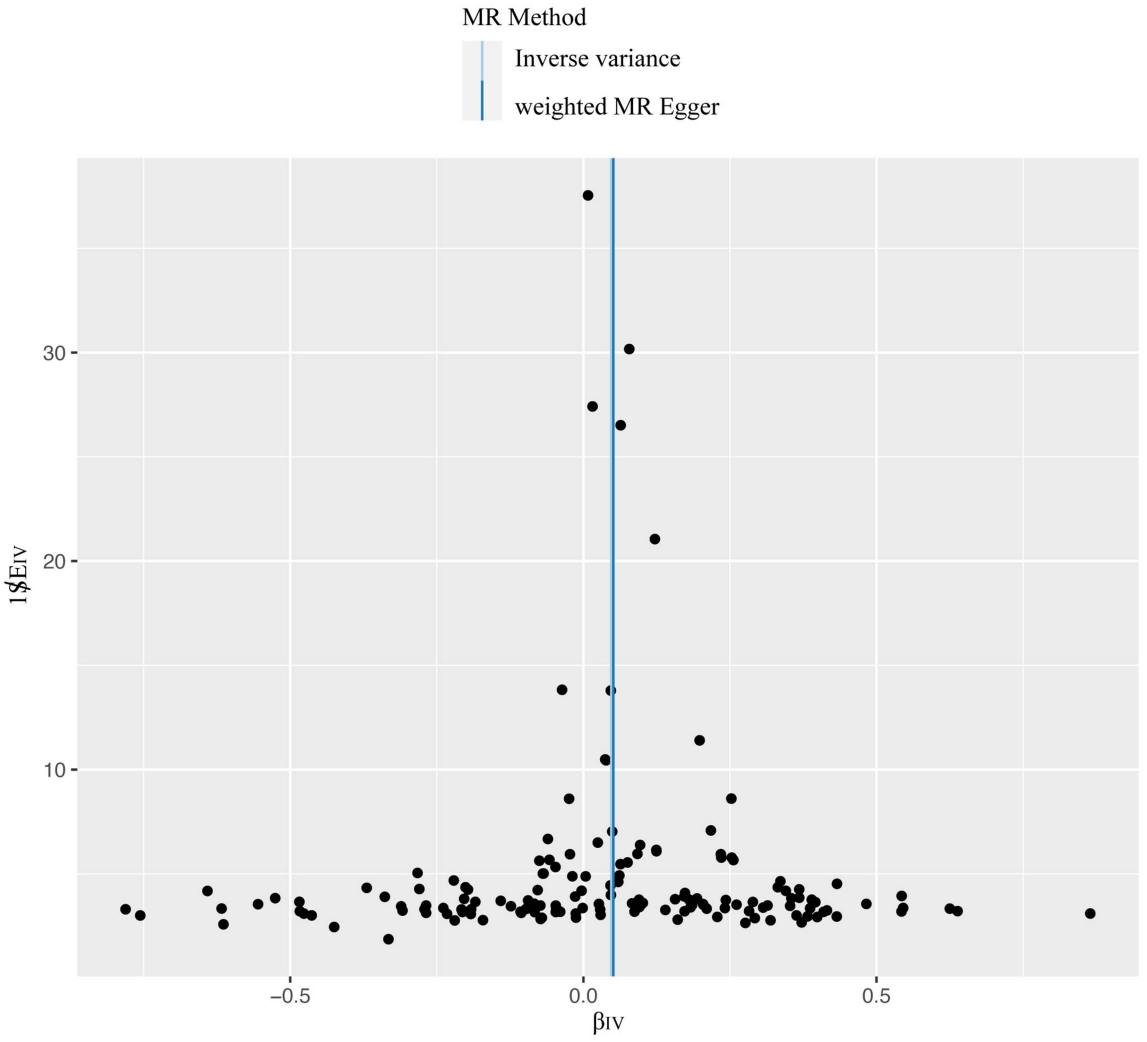
The overall heterogeneity test was conducted to assess the impact of T1DM on candidiasis. SNPs are depicted by black points, and their distribution is evenly spread around the IVW and MR-Egger line. T1DM, type 1 diabetes mellitus; SNP, single-nucleotide polymorphism; IVW, inverse variance weighting.

**Figure 7:**
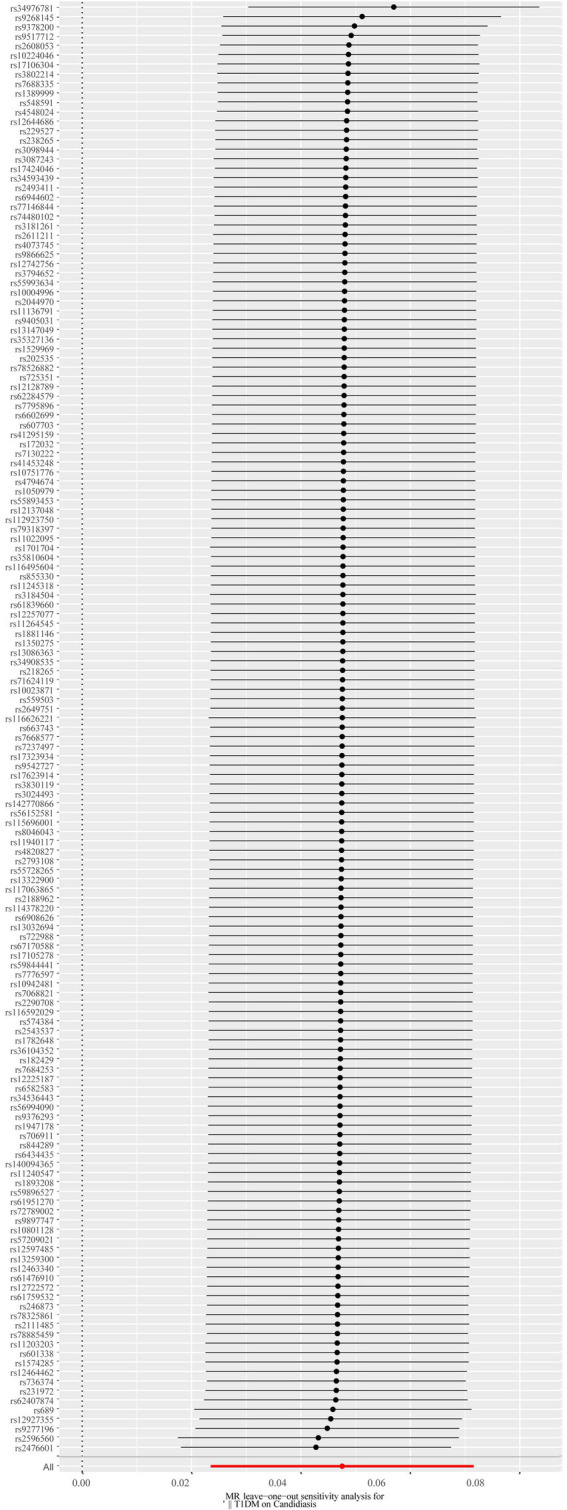
The forest plot of the leave-one-out analysis. The red data point’s position above 0 suggests a positive outcome. The black dots are situated to the right of the invalid line, suggesting that the removal of any of the SNPs will have minimal impact on the overall results. SNP, single-nucleotide polymorphism.

A total of 153 SNPs were identified as IVs for this study, each demonstrating robustness with F-statistics exceeding 10 (ranging from 18.09 to 1772.13), which underscores their suitability for evaluating the relationship between T1DM and candidiasis. All SNP details are provided in Supplementary Table S1. The GWAS data for T1DM were visually represented on a Manhattan plot (Figure 2). Figure 3 illustrates the individual impact of each SNP on the risk of developing candidiasis. Various analytical methods consistently demonstrated an increased risk of candidiasis among T1DM patients: the IVW method showed an OR of 1.05 (95% CI 1.02–1.07, *p* < 0.001), MR-Egger yielded an OR of 1.05 (95% CI 1.02–1.09, *p* = 0.003), the weighted median method reported an OR of 1.05 (95% CI 1.01–1.09, *p* = 0.025), and the weighted mode method indicated an OR of 1.04 (95% CI 1.01–1.07, *p* = 0.01), with all tests showing consistent beta directions (Table 1; Figure 4). Figure 5 presents a scatter plot which validates the increased risk of candidiasis in T1DM patients. The univariate MR analysis indicated no significant heterogeneity, as shown by the MR-Egger (*p* = 0.17) and IVW (*p* = 0.18) tests. Furthermore, no significant pleiotropy was detected, with the MR-Egger intercept revealing no substantial influence (intercept = −0.00099; *p* = 0.76). The MR-PRESSO test confirmed the absence of outliers (Table 2), and the symmetry of selected SNPs was clear in the funnel plot (Figure 6). Additionally, the scatter plot visually reinforced the causal link between T1DM and candidiasis. The forest plot displayed effect sizes for individual SNPs on the risk of candidiasis, confirming the causal relationship. Notably, the “Leave-one-out” plot analysis demonstrated that no single SNP significantly influenced the estimated causal association (Figure 7).

**Table 1:** Results of MR analyses.

Outcome	MR methods	SNP number	OR(95%CI)	*p* value
Candidiasis	MR Egger	153	1.05 (1.02–1.09)	0.003
	Weighted median	153	1.05 (1.01–1.09)	0.025
	Inverse variance weighted	153	1.05 (1.02–1.07)	<0.001
	Simple mode	153	1.02 (0.94–1.11)	0.586
	Weighted mode	153	1.04 (1.01–1.07)	0.01
Pneumocystosis	MR Egger	153	1.07 (0.96–1.18)	0.24
	Weighted median	153	1.09 (0.97–1.22)	0.17
	Inverse variance weighted	153	1.03 (0.95–1.11)	0.50
	Simple mode	153	1.13 (0.84–1.52)	0.42
	Weighted mode	153	1.09 (0.99–1.20)	0.093
Aspergillosis	MR Egger	147	0.89 (0.71–1.10)	0.28
	Weighted median	147	0.99 (0.76–1.30)	0.95
	Inverse variance weighted	147	0.94 (0.80–1.10)	0.43
	Simple mode	147	0.91 (0.51–1.63)	0.75
	Weighted mode	147	0.91 (0.74–1.11)	0.35
Other mycoses	MR Egger	153	1.04 (0.81–1.32)	0.76
	Weighted median	153	1.04 (0.78–1.39)	0.80
	Inverse variance weighted	153	1.09 (0.91–1.30)	0.35
	Simple mode	153	1.08 (0.55–2.12)	0.83
	Weighted mode	153	1.14 (0.9–1.44)	0.28

MR, Mendelian randomization; SNP, single-nucleotide polymorphisms; OR, odd ratio; CI, confidence intervals; MR Egger, Mendelian randomization-egger regression.

**Table 2:** Sensitive analysis of T1DM on outcomes.

Outcome	Heterogeneity		Pleiotropy		Outliers (MR-PRESSO)
	IVW	MR Egger	Intercept	*p* value	
Candidiasis	0.18	0.17	−0.00099	0.76	No
Pneumocystosis	0.95	0.95	−0.0102	0.31	No
Aspergillosis	0.59	0.58	0.016	0.45	No
Other mycoses	0.62	0.61	0.013	0.58	No

IVW, inverse variance weighted; MR-PRESSO, Mendelian randomization pleiotropy residual sum and outlier; MR Egger, Mendelian randomization-egger regression.

### 3.2 MVMR analyses the causal effect of T1DM onset candidiasis

The MVMR-IVW estimates revealed a significant causal relationship between T1DM and candidiasis, even after adjusting for BMI, HbA1c, and counts of neutrophils, lymphocytes, and monocytes (OR=1.08; 95% CI, 1.03–1.13; *p*=0.0006). The analysis showed no heterogeneity (MR-IVW, *p*=0.30; MR-Egger, *p*=0.29) and no potential pleiotropy (mv-pleiotropy, p=0.30). The robustness of these instruments is underscored by an F-statistic of 14.98. Further adjustments for immune cell classifications, including CD4 regulatory T cells, CD8+ T cells, CD4+ T cells, NK T cells, and B cell absolute counts, reinforced the causal link between T1DM and candidiasis. The adjusted MVMR-IVW analyses yielded an OR of 1.04 (95% CI, 1.02–1.06; *p*=0.0002), with no detected heterogeneity (MR-IVW, *p*=0.77; MR-Egger, *p*=0.75) or pleiotropy (mv-pleiotropy, *p*=0.95). The strength of this model is highlighted by an F-statistic of 86.73, suggesting that the instrumental variables are highly relevant and the findings are statistically sound.

### 3.3 Exploration of the causal effect of T1DM onset pneumocystosis, aspergillosis, and other mycoses

In the MR analyses exploring the effects of T1DM on pneumocystosis, aspergillosis, and other mycoses, we utilized 153, 147, and 153 SNPs as IVs, respectively. All IVs demonstrated robustness with F-statistics exceeding 10, confirming their suitability for the analysis. Detailed SNP information for each condition can be found in Supplementary Tables S2–S4. Our findings indicated no causal relationship between T1DM and pneumocystosis, aspergillosis, or other mycoses when analyzed using the IVW method. Similar results were consistent across additional MR methods as detailed in Table 1. Furthermore, the analyses showed no signs of pleiotropy or heterogeneity in the relationship between T1DM and these mycoses, as shown in Table 2. Visual aids such as forest plots, scatter plots, funnel plots, and leave-one-out plots for each mycosis have been detailed in Supplementary Figures S1–S12, respectively. These visualizations further corroborate the robustness and transparency of our findings, illustrating the analytical process and outcomes comprehensively.

## 4 Discussion

Our comprehensive MR investigation revealed a significant causal association between T1DM and candidiasis. Cochran’s Q test indicated no heterogeneity, a finding further supported by the symmetrical distribution observed in the funnel plot. Moreover, both the MR PRESSO and MR Egger tests detected no evidence of horizontal pleiotropy. Figure 5 illustrates that all MR test results pointed in the same positive direction, confirming a consistent correlation between T1DM and the occurrence of candidiasis. Figure 6 confirms the absence of heterogeneity in the results, while Figure 7 demonstrates that no single SNP significantly impacts the estimated causal association. These sensitivity analyses underscore the robustness of our results. Further, in the MVMR analyses, the significant association between T1DM and candidiasis persisted even after adjustments for BMI, HbA1c, and counts of neutrophils, lymphocytes, and monocytes. Additionally, our study indicates that T1DM may not be implicated in the development of certain other mycoses, including aspergillosis and pneumocystis, suggesting a more specific immunological interaction with candidiasis. This nuanced understanding could guide more targeted approaches in the management and prevention of mycoses in patients with T1DM.

The main findings of our research align with prior investigations in the field. In a study involving 32 young females with T1DM, Candida species were detected in 52.5% of cases (21), significantly higher than the control group’s rate of 18.2%. Prevalent species included *C. albicans* (72.7%), *Candida glabrata* (22.7%), *Candida tropicalis* (2.3%), and *Candida parapsilosis* (2.3%) (22). In a study, a 75.5% prevalence of gastrointestinal candidiasis was reported among patients diagnosed with T1DM, while other investigations indicated rates ranging from 2.5 to 9.7% (23). Moreover, elevated HbA1c levels were associated with oral candidiasis and gingivitis in pediatric T1DM patients. Candidiasis affecting skin folds and mucous membranes, have been documented in more than 5% of patients (24). Candida species are the primary fungal colonizers of the urinary tract in diabetic individuals (25), with infection occurring in the presence of symptoms or pyuria. Moreover, investigations into candidemia among intensive care unit patients underscore diabetes mellitus as a notable risk factor for candidemia onset. Dissemination of candidemia to the lungs can lead to secondary pulmonary candidiasis (26).

A study investigated the association between diabetes and both the isolation and infection with *aspergillus species* (27). In a study by Sun et al. (28) in 2017, it was highlighted that diabetes mellitus had a prevalence of 18.4% among 407 patients diagnosed with invasive pulmonary aspergillosis (IPA), ranking as the second most common underlying condition after hematological malignancies. Additionally, Xu et al. (29) observed that 30% of patients with chronic obstructive pulmonary disease (COPD) who developed IPA also had diabetes, contrasting with only 6.7% of COPD patients without aspergillosis having diabetes (*p*<0.05). Collectively, these studies suggest that diabetes mellitus may heighten the likelihood of developing invasive pulmonary aspergillosis, particularly when combined with other risk factors. Nonetheless, larger epidemiological studies are needed to validate the association between diabetes and aspergillosis (30), and the current research does not genetically support a higher predisposition to aspergillosis in patients with T1DM.

A comprehensive review of current literature highlights several factors contributing to the occurrence of pneumocystis pneumonia (PCP). Advanced age, lymphopenia, the administration of high-dose steroids, triple immunosuppression, and specific comorbidities such as chronic obstructive pulmonary disease (COPD) have been identified as influential factors. Furthermore, epidemiological research conducted in Japan has shed light on prevalent comorbidities associated with PCP among adults. This study revealed that hematologic malignancies (31%) and diabetes (30%) were the most commonly observed comorbidities in individuals diagnosed with PCP. These findings underscore the importance of considering these risk factors in clinical assessments and management strategies for PCP (31). Studies have indicated that older age, gender, type of transplant, cytomegalovirus infection, allograft rejection, and immunosuppression are significant variables that increase the risk of developing pneumocystis pneumonia post-transplant (32). However, these studies have primarily stemmed from studies conducted in individual medical centers. Reports suggest that around 1 to 2% of patients with rheumatologic disorders (33), particularly those undergoing immunosuppressive treatment, have developed PCP. Overall, the limited direct research on the correlation between diabetes mellitus and pneumocystis pneumonia mainly consists of retrospective epidemiological investigations from single-center studies with numerous confounding factors. Our study proposes that T1DM may not have a genetic association with pneumocystis pneumonia, a hypothesis that warrants further confirmation through prospective studies.

In Europe and the United States, *Trichinella* infections primarily affect patients undergoing chemotherapy for hematologic malignancies, solid organ transplants, or bone marrow transplants. Conversely, in Asian countries, diabetic patients experience a higher incidence of *Trichinella* infections. *Cryptococcal* infections, on the other hand, are most prevalent in immunocompromised patients. In a retrospective study examining *cryptococcosis* in patients with diabetes mellitus, it was found that 62% of the patients had poor glycemic control. This observation suggests a potential relationship between blood glucose levels and the occurrence of *cryptococcal* infections (34). Similarly, we found no exceptions to their association with T1DM genetics. High glucose and acidic environments facilitate the growth and proliferation of molds. Iron ions are also essential for mold growth. In diabetic ketoacidosis, when serum pH decreases due to acidosis, the transport capacity of transferrin for iron is inhibited, leading to an increase in serum free iron, which promotes mold growth. The results of a prospective multicenter study showed that among 50 patients with pulmonary trichinosis (clinically diagnosed or above), 57% had poorly controlled diabetes, 18% had ketoacidosis, and observations highlighted diabetes as an autonomous risk factor for the initiation of pulmonary trichinosis (35, 36). However, there is a lack of separate GWAS databases for *Trichoderma* and *Cryptococcus*, so separate Mendelian randomization studies are not possible.

Elevated blood sugar levels have adverse effects on the immune system, altering tissues, skin, and blood circulation, thereby increasing susceptibility to infections. Specifically, high blood sugar levels and inadequate insulin levels suppress vital components of the body’s innate immune response to pathogens and impede the production of pro-inflammatory cytokines. Studies have shown that individuals with Type 1 diabetes mellitus exhibit reduced secretion of interleukin 1 and interleukin 6 by mononuclear cells and monocytes (7, 37). Additionally, chronic hyperglycemia impairs the mobilization, chemotaxis, and phagocytosis of polymorphonuclear leukocytes, further compromising the body’s ability to combat infections (38, 39). Elevated blood sugar levels, also known as hyperglycemia, disrupt antimicrobial activity through various mechanisms. These include increased apoptosis, diminished mobility of polymorphonuclear cells across endothelial layers, and inhibition of glucose 6-phosphate dehydrogenase (40). Moreover, individuals diagnosed with T1DM demonstrate compromised complement activity, marked by diminished C4 levels, impaired cytokine production, and dysfunction of polymorphonuclear cells (37).

The design of the MR study, aimed at mitigating confounding factors and addressing reverse causality inherent in epidemiological studies, stands out as a crucial strength of this research. Furthermore, a comprehensive exploration of the causal links between T1DM and fungal infections was meticulously conducted. All selected IVs exhibited robustness, as indicated by F-statistics exceeding 10. Moreover, the absence of pleiotropy provided further support for the accuracy of our findings. Upon establishing a causal relationship between T1DM and candidiasis, three separate MVMR analyses were performed to validate the results by adjusting for BMI and HbA1c. Notably, MVMR Egger yielded reliable estimates despite significant pleiotropy, with no observed heterogeneity. The consistency across outcomes obtained through the three methodologies underscores the credibility of our findings. It is important to acknowledge that the datasets used for exposure and outcome variables primarily comprised European populations.

The current study presents several advancements over previous research. Most notably, it is the first MR study to elucidate the genetic causal links between T1DM and mycoses. A major strength of this approach is the MR design itself, which significantly reduces the risk of reverse causality and confounding factors affecting the results. By focusing on populations of the same ethnicity, this study also effectively minimizes racial and ethnic disparities that could skew findings. However, the study is not without limitations. Firstly, it’s important to recognize that the results may still be influenced by other potential confounders not accounted for in the analysis. Secondly, the reliance on Finnish and EBI databases could introduce bias and limit the generalizability of the findings. Additionally, the absence of GWAS data for *mucormycosis* and *cryptococcosis* in the EBI database compelled us to rely solely on data from the FinnGen database for these and other mycoses. Lastly, setting a *p*-value threshold of 5×10^^−6^ may increase the risk of false positives in our findings.

In conclusion, our MR analysis supports a genetic predisposition for increased susceptibility to candidiasis in individuals with T1DM. However, the study did not establish a causal relationship between T1DM and other fungal infections, such as aspergillosis, pneumocystis, and other types of mycoses. This specificity in the interaction between T1DM and candidiasis may inform future research and clinical approaches.

## Data availability statement

The datasets presented in this study can be found in online repositories. The names of the repository/repositories and accession number(s) can be found in the article/Supplementary material.

## Ethics statement

Ethical review and approval was not required for the study on human participants in accordance with the local legislation and institutional requirements. Written informed consent from the patients/participants or patients/participants’ legal guardian/next of kin was not required to participate in this study in accordance with the national legislation and the institutional requirements.

## Author contributions

XC: Data curation, Formal analysis, Resources, Writing – original draft, Writing – review & editing. CC: Data curation, Formal analysis, Writing – original draft. MW: Investigation, Writing – review & editing. SW: Supervision, Writing – review & editing. HJ: Resources, Writing – review & editing. ZL: Methodology, Project administration, Writing – review & editing. YY: Data curation, Funding acquisition, Resources, Software, Validation, Writing – original draft, Writing – review & editing. BL: Data curation, Project administration, Supervision, Validation, Visualization, Writing – original draft, Writing – review & editing.
