# Supplementary material for: Causal relationship between type 1 diabetes mellitus and mycoses: a Mendelian randomization study
Source: Front Med (Lausanne). 2024 Jun 14;11:1408297. doi: 10.3389/fmed.2024.1408297 (PMC11211379; doi:10.3389/fmed.2024.1408297)

**Fig.S1.** The forest plot depicts the impact of each SNP on pneumocystosis.Black lines represent the effect of an individual SNP, while a red line signifies the causal estimation using all ivs. If the solid line is positioned entirely to the left of 0, it suggests that T1DM may reduce the likelihood of candidiasis based on this SNP. Conversely, it implies that T1DM might potentially increase the risk of candidiasis. An intersection of the solid line with 0 indicates an insignificant result.


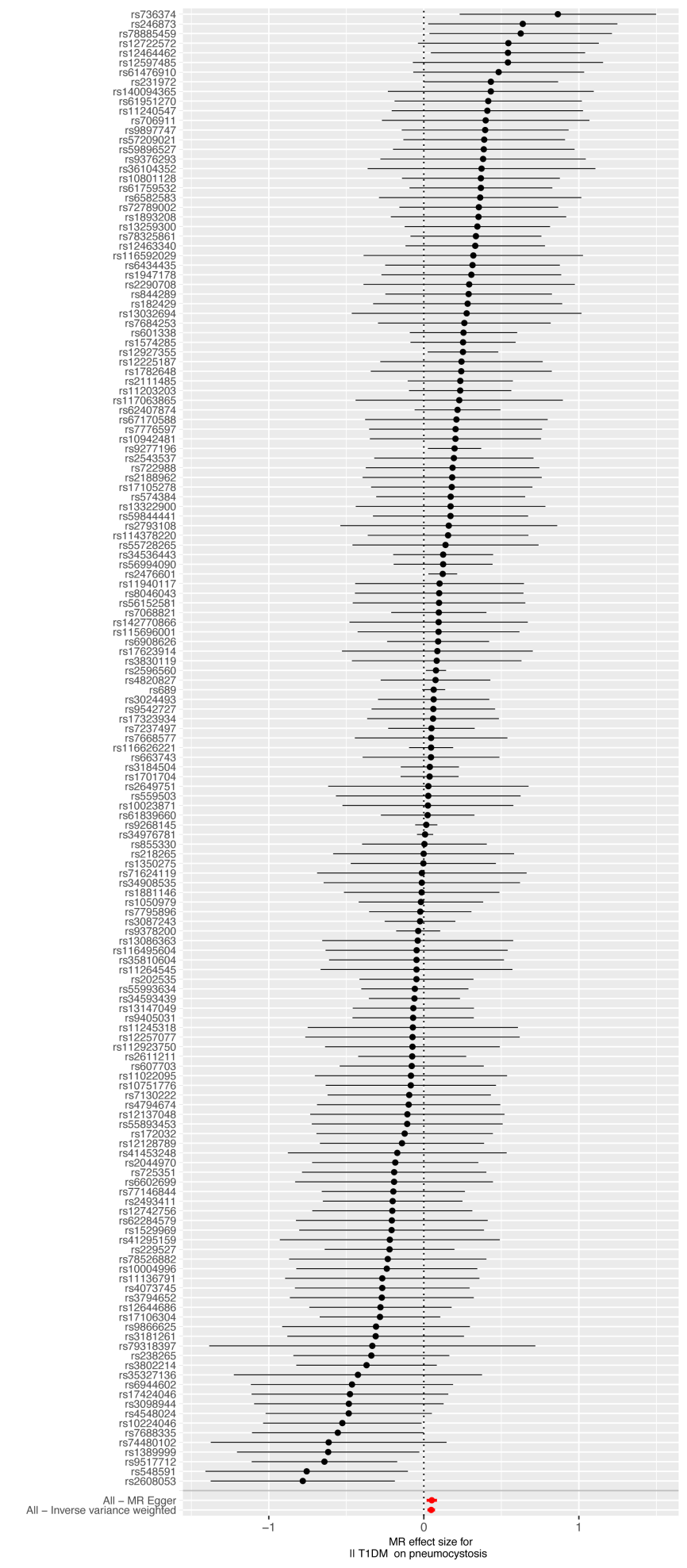


**Fig.S2.** The forest plot depicts the impact of each SNP on aspergillosis.Black lines represent the effect of an individual SNP, while a red line signifies the causal estimation using all ivs. If the solid line is positioned entirely to the left of 0, it suggests that T1DM may reduce the likelihood of candidiasis based on this SNP. Conversely, it implies that T1DM might potentially increase the risk of candidiasis. An intersection of the solid line with 0 indicates an insignificant result.


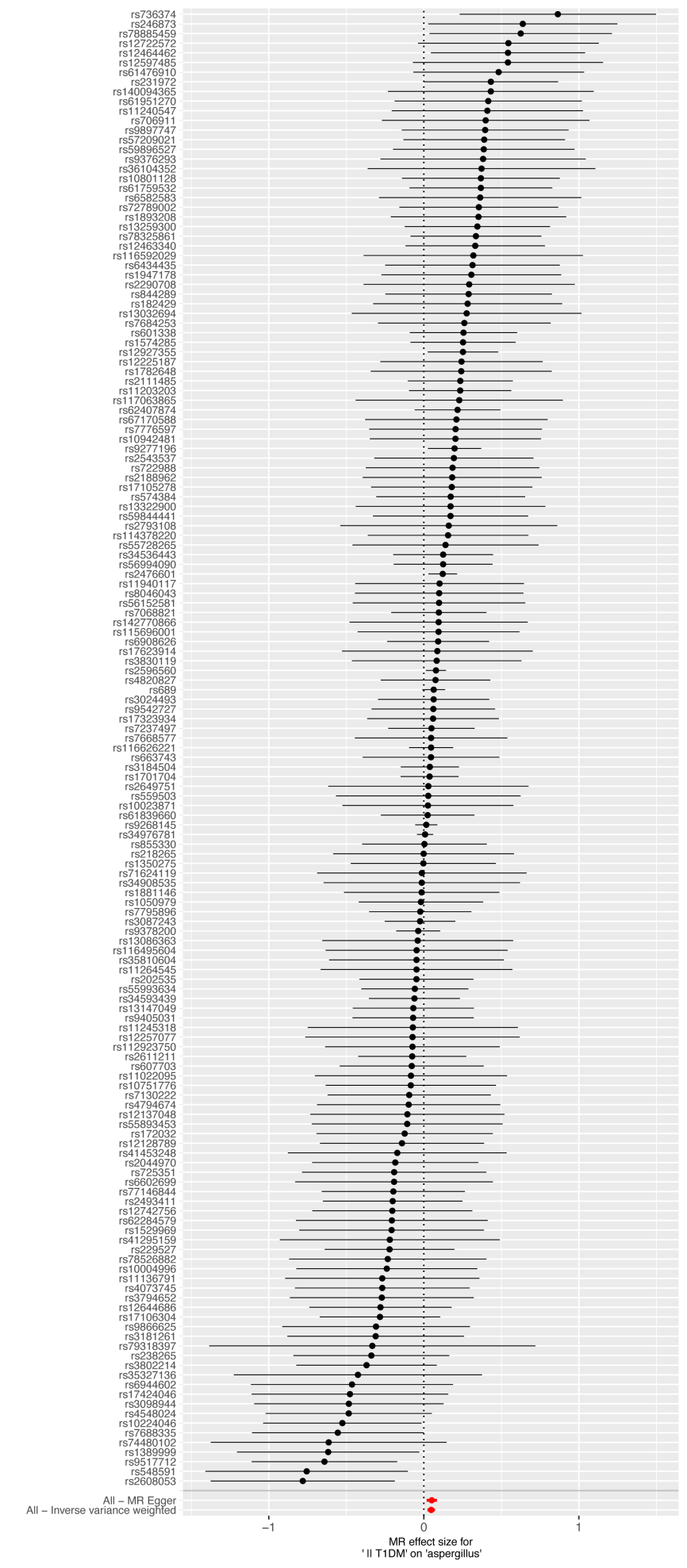


**Fig.S3.** The forest plot depicts the impact of each SNP on other mycoses.Black lines represent the effect of an individual SNP, while a red line signifies the causal estimation using all ivs. If the solid line is positioned entirely to the left of 0, it suggests that T1DM may reduce the likelihood of candidiasis based on this SNP. Conversely, it implies that T1DM might potentially increase the risk of candidiasis. An intersection of the solid line with 0 indicates an insignificant result.


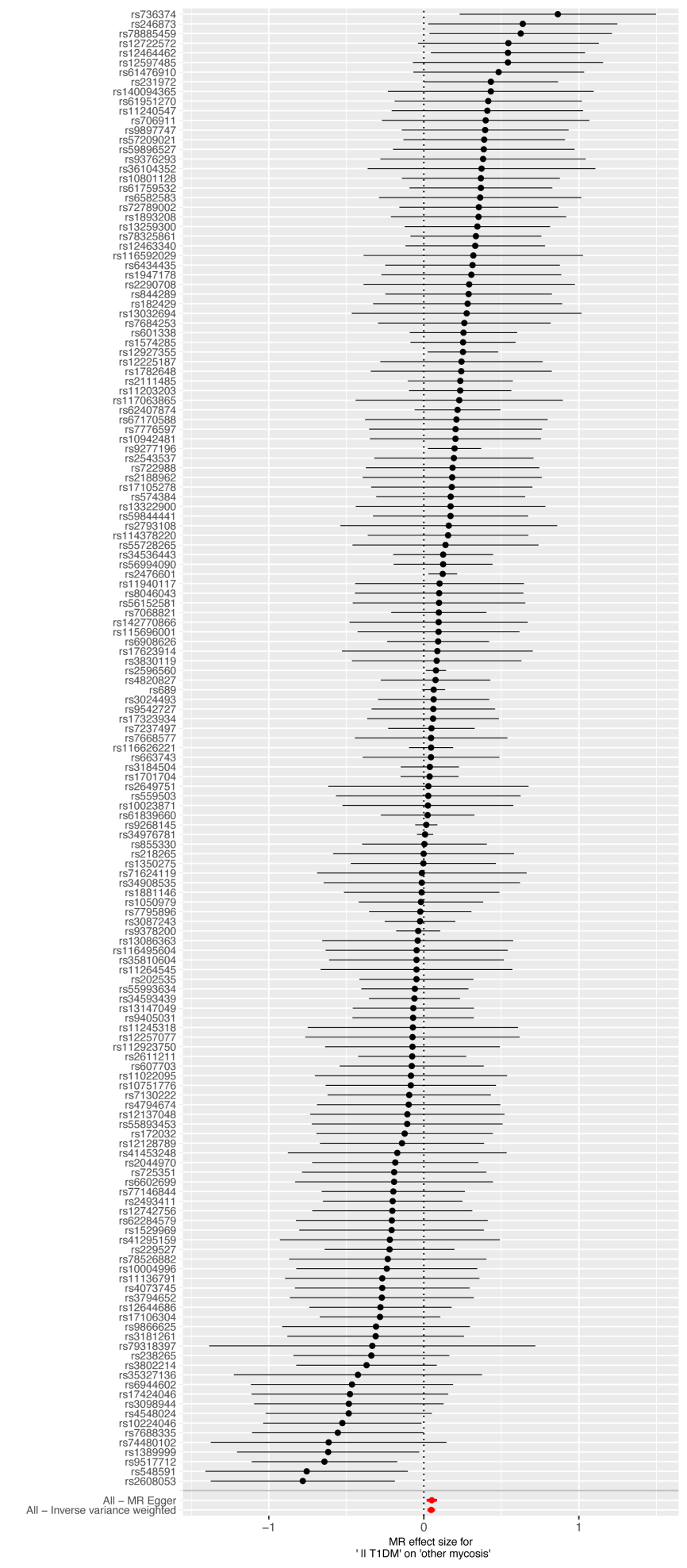


**Fig.S4.**The scatter plot of casual effect of T1DM on pneumocystis. Black data points represent ivs, with the horizontal axis indicating the influence of SNPs on T1DM, and the vertical axis indicating the influence of SNPs on candidiasis occurrence. Colored lines depict the results of MR analysis utilizing five different methods.


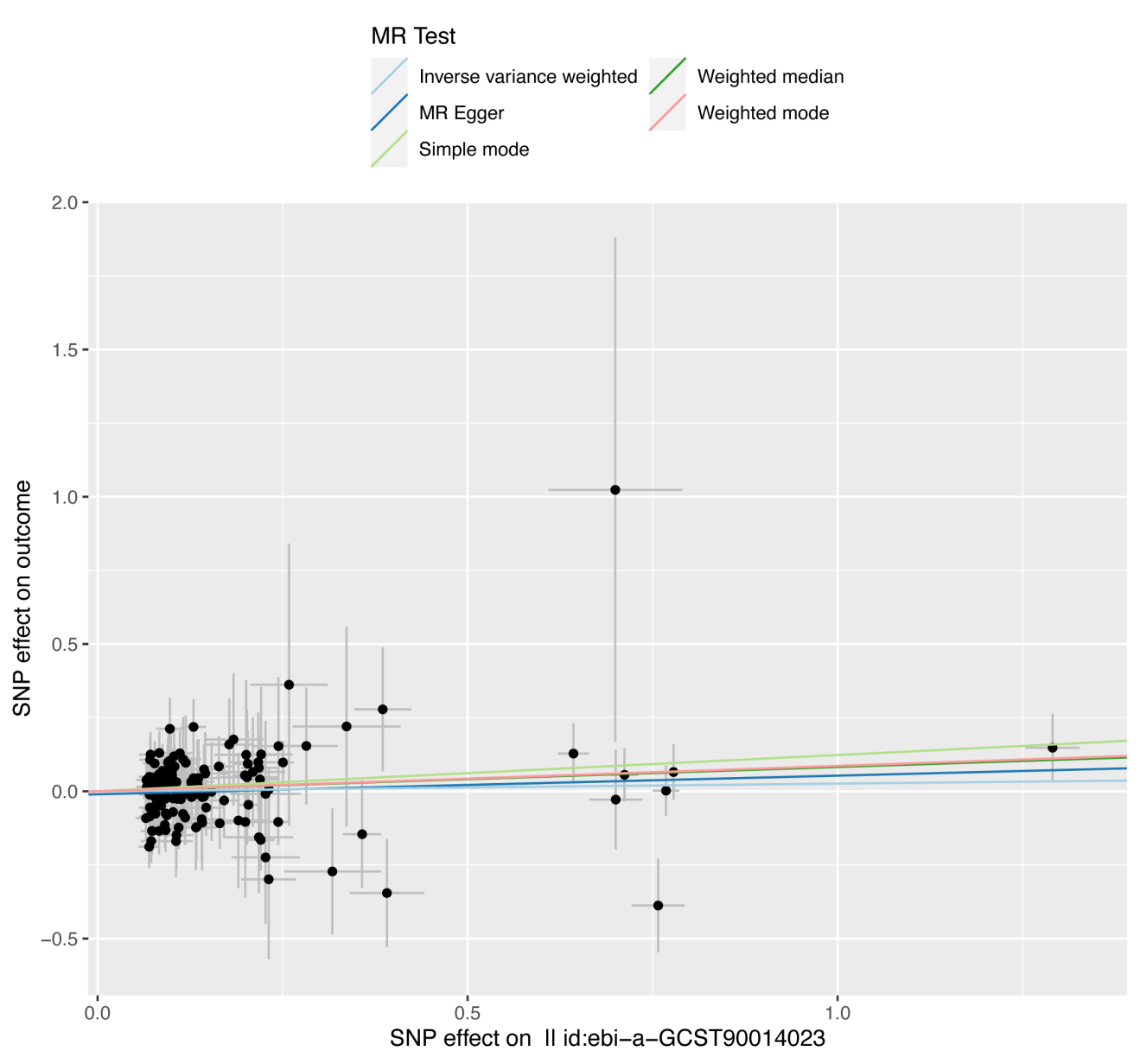


**Fig.S5.**The scatter plot of casual effect of T1DM on aspergillosis. Black data points represent ivs, with the horizontal axis indicating the influence of SNPs on T1DM, and the vertical axis indicating the influence of SNPs on candidiasis occurrence. Colored lines depict the results of MR analysis utilizing five different methods.


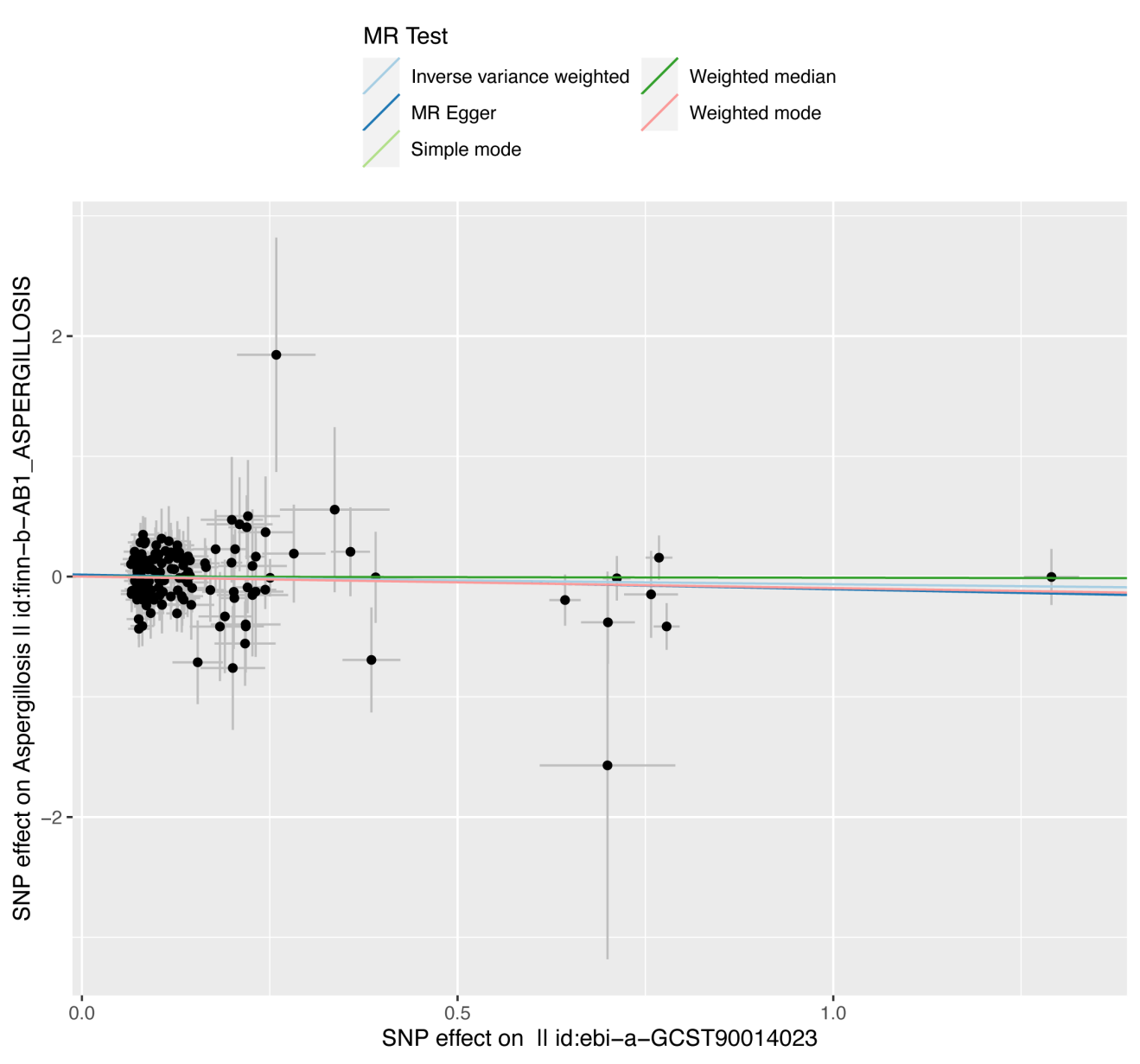


**Fig.S6.**The scatter plot of casual effect of T1DM on other mycoses. Black data points represent ivs, with the horizontal axis indicating the influence of SNPs on T1DM, and the vertical axis indicating the influence of SNPs on candidiasis occurrence. Colored lines depict the results of MR analysis utilizing five different methods.


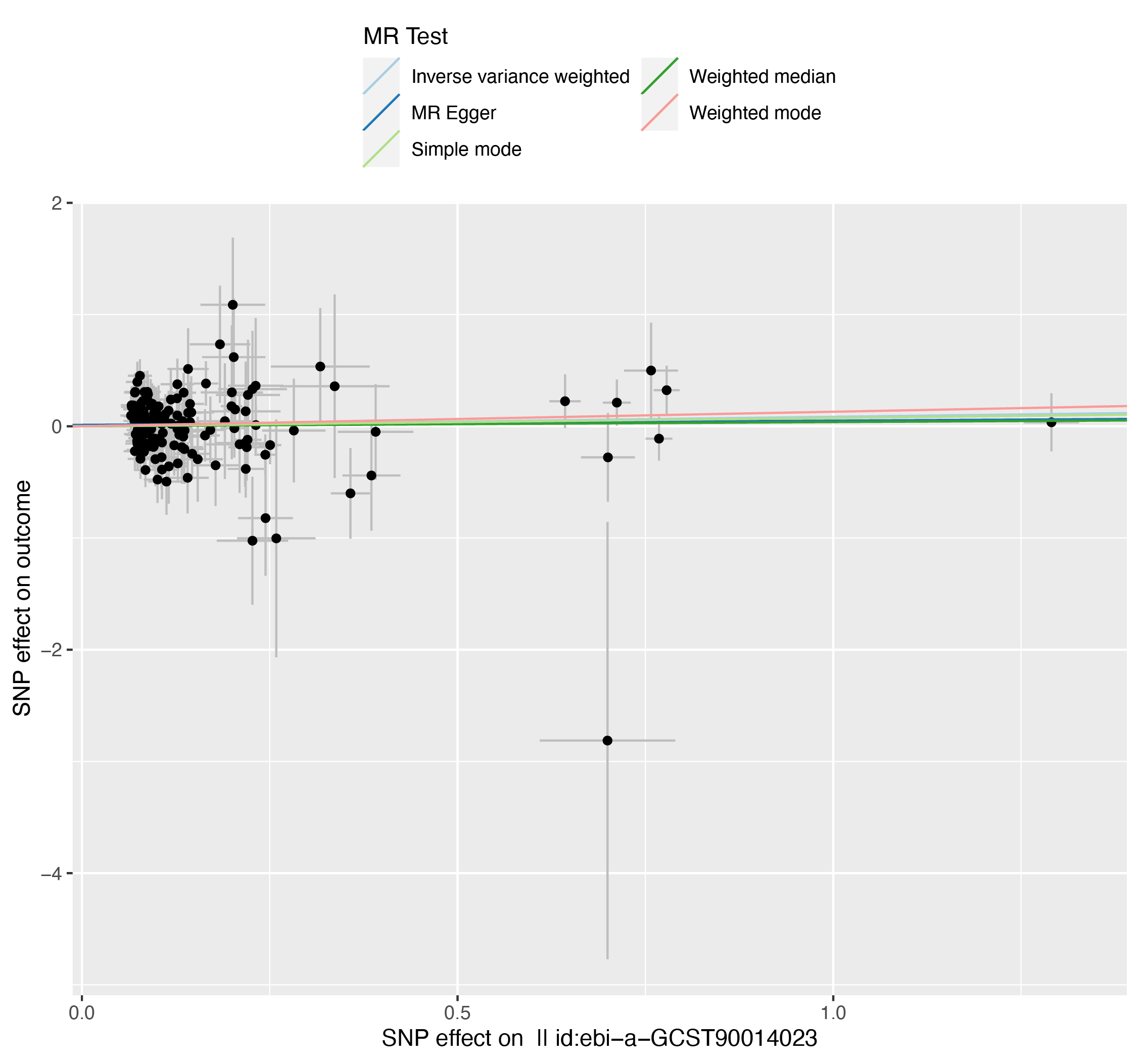


**Fig.S7.** The overall heterogeneity test was conducted to assess the impact of T1DM on pneumocystis. SNPs are depicted by black points, and their distribution is evenly spread around the IVW and MR-Egger line.


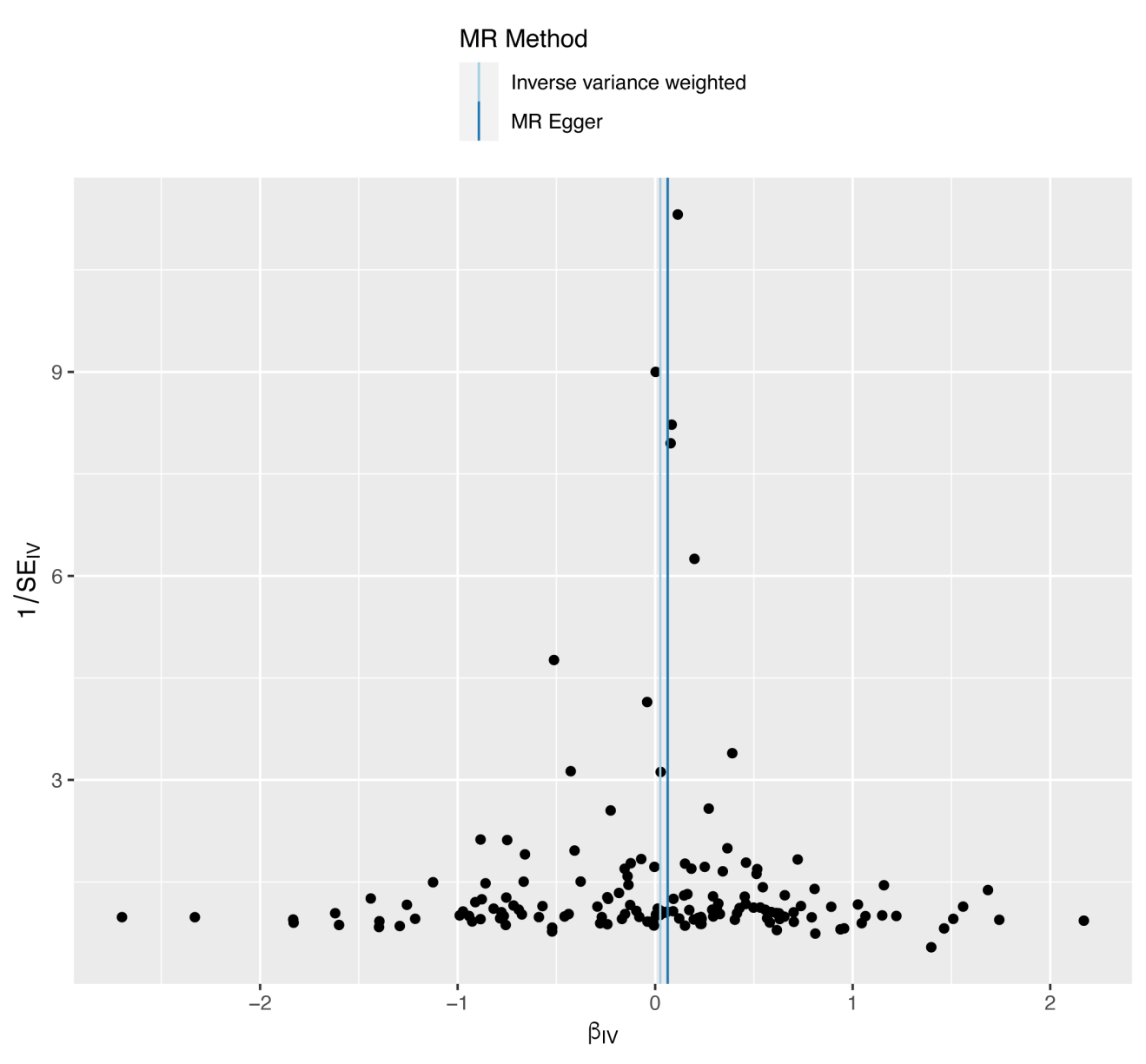


**Fig.S8.** The overall heterogeneity test was conducted to assess the impact of T1DM on aspergillosis SNPs are depicted by black points, and their distribution is evenly spread around the IVW and MR-Egger line.


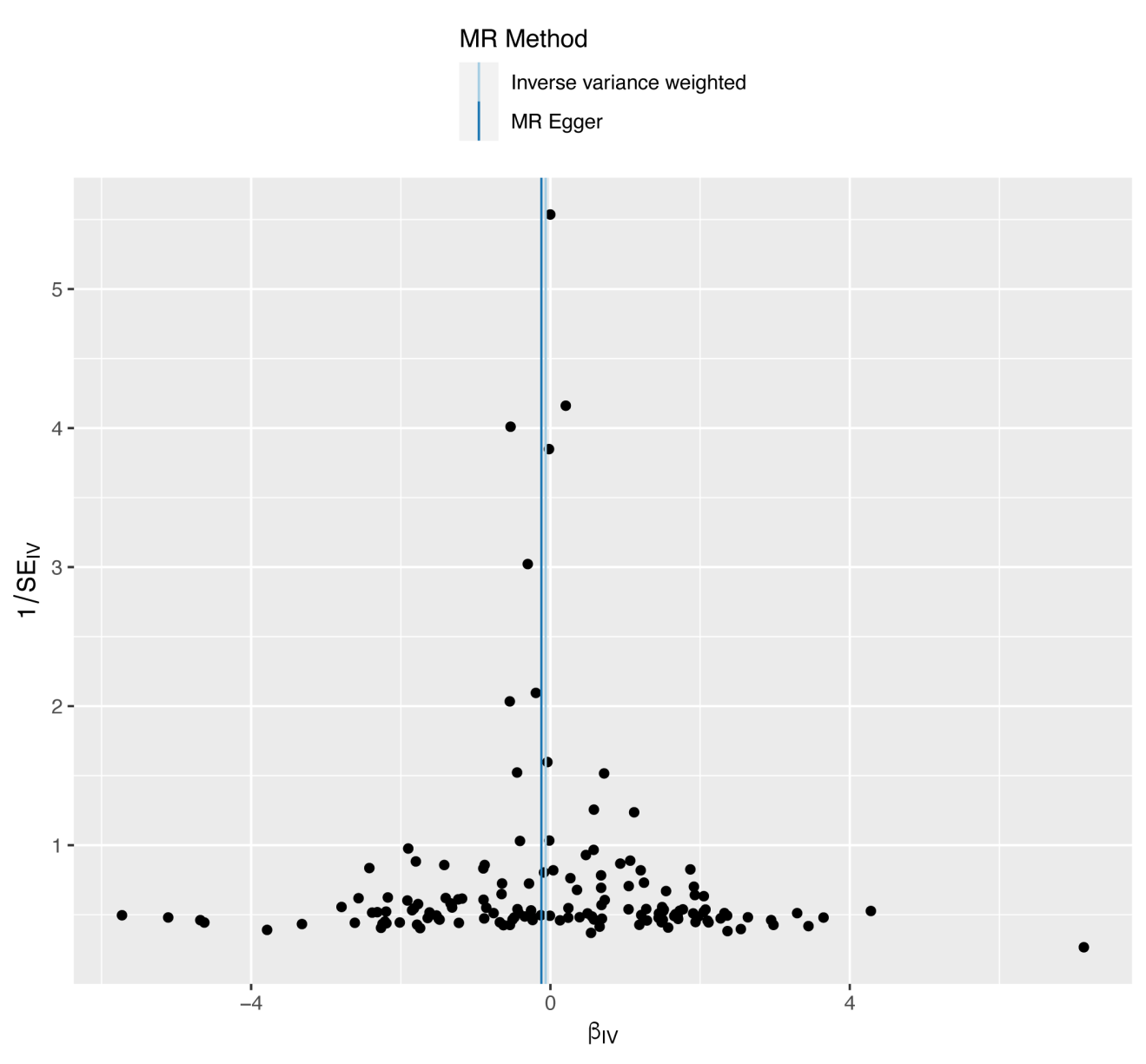


**Fig.S9.** The overall heterogeneity test was conducted to assess the impact of T1DM on other mycoses SNPs are depicted by black points, and their distribution is evenly spread around the IVW and MR-Egger line.


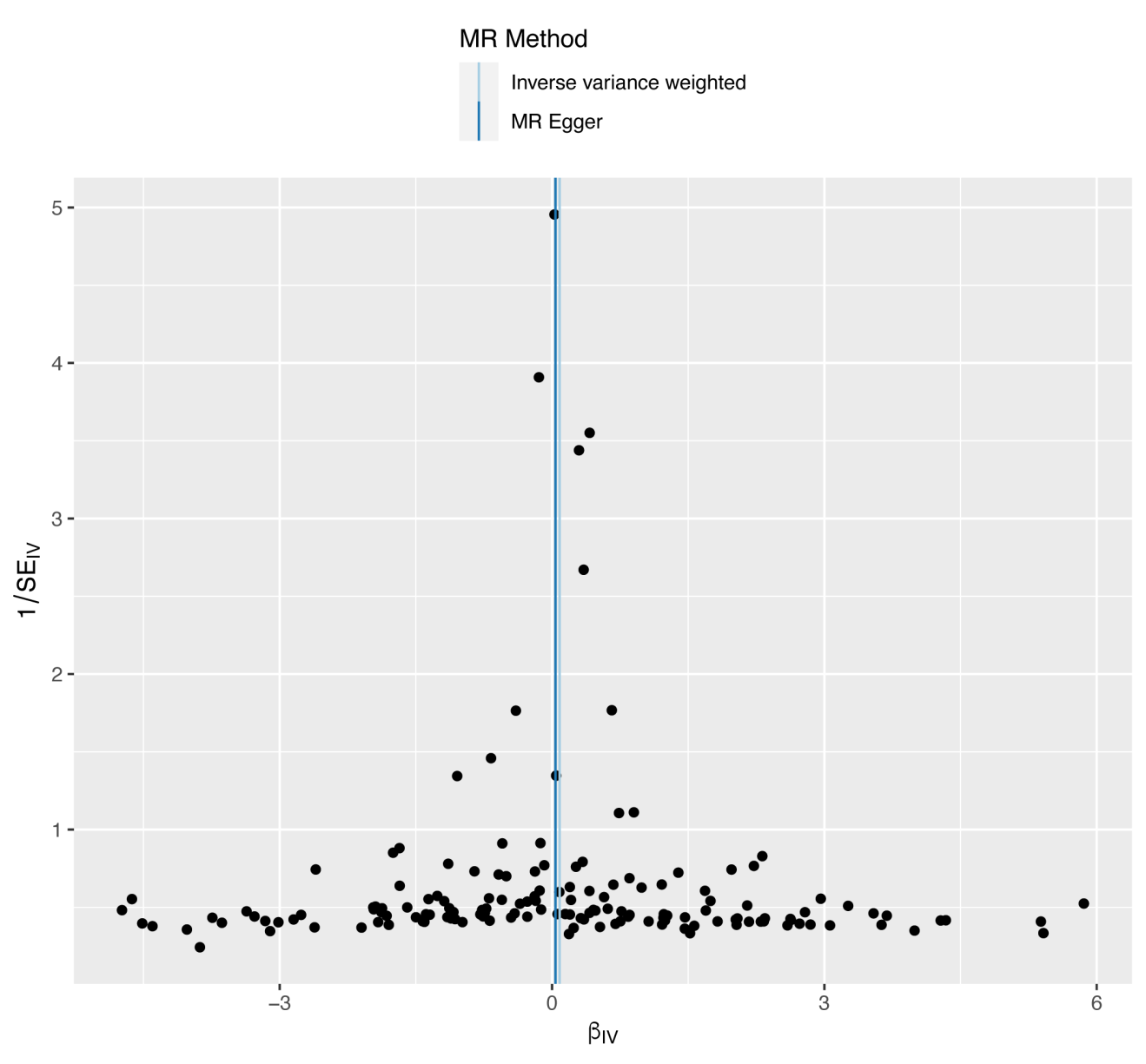


**Fig.S10.** The forest plot of the leave-one-out analysis on pneumocystis. The red data point's position above 0 suggests a positive outcome.The black dots are situated to the right of the invalid line, suggesting that the removal of any of the SNPs will have minimal impact on the overall results.


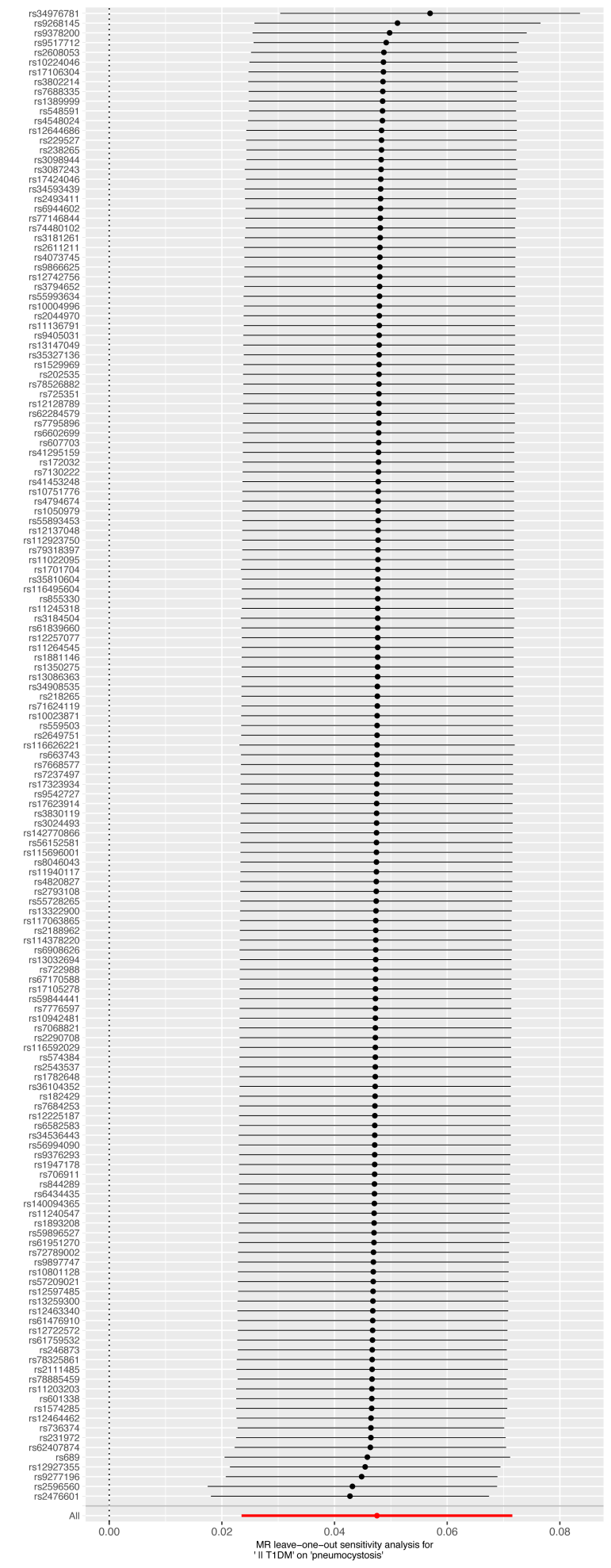


**Fig.S11.** The forest plot of the leave-one-out analysis on aspergillosis. The red data point's position above 0 suggests a positive outcome.The black dots are situated to the right of the invalid line, suggesting that the removal of any of the SNPs will have minimal impact on the overall results.


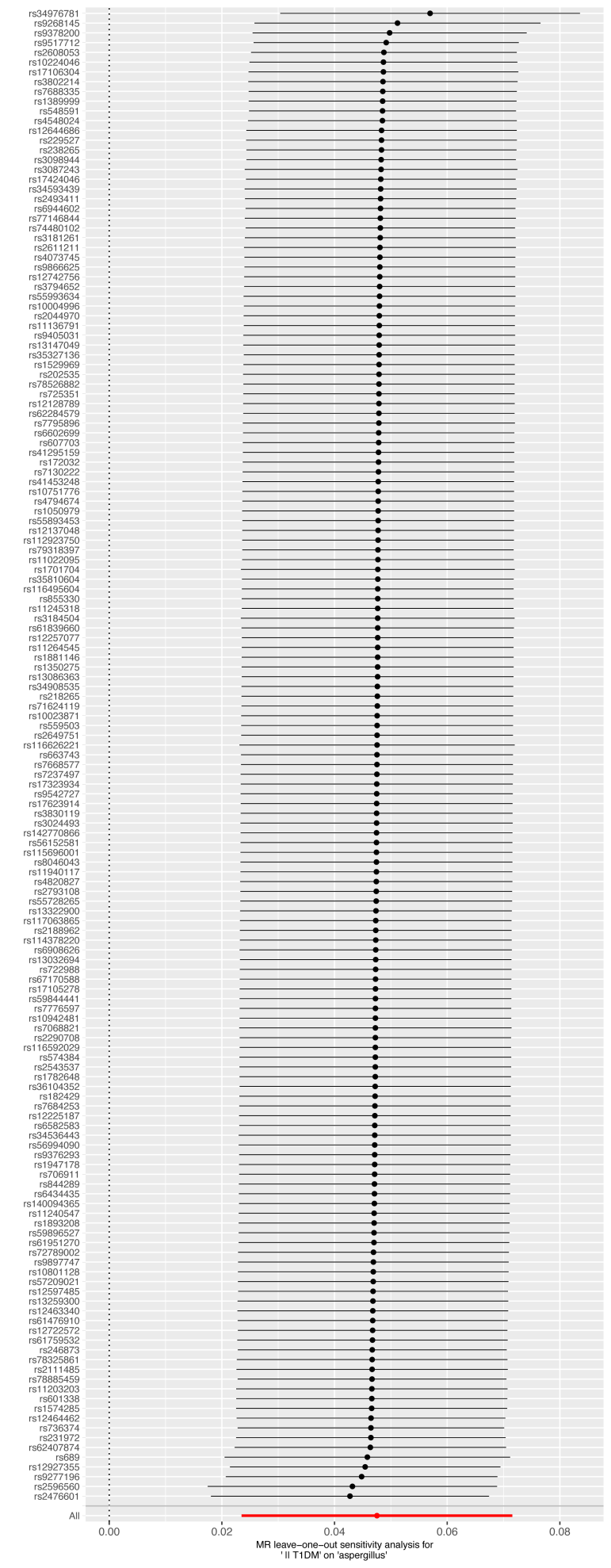


**Fig.S12.** The forest plot of the leave-one-out analysis on other mycoses. The red data point's position above 0 suggests a positive outcome.The black dots are situated to the right of the invalid line, suggesting that the removal of any of the SNPs will have minimal impact on the overall results.


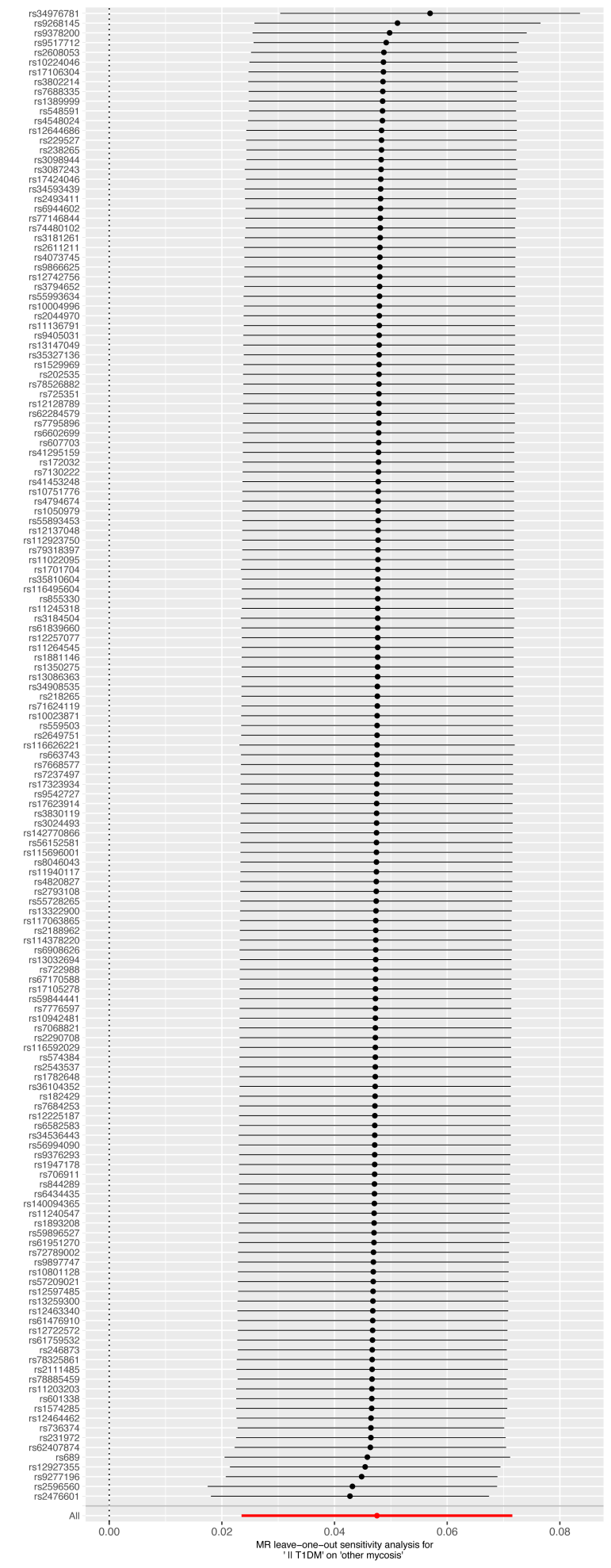

Supplement: Supplementary file 1 [file Data_Sheet_1.docx]
